# Protocol to detect smooth muscle actin-alpha and measure oxidative damage in neonatal mouse intestine

**DOI:** 10.1016/j.xpro.2022.101524

**Published:** 2022-07-09

**Authors:** Shing Hu, Carolyn S. Sevier, Natasza A. Kurpios

**Affiliations:** 1 Department of Molecular Medicine, College of Veterinary Medicine, Cornell University, Ithaca, NY 14853, USA

**Keywords:** Cell Biology, Developmental biology, Molecular Biology

## Abstract

This protocol describes how to characterize α-Smooth muscle actin (αSMA) spatiotemporal expression during mouse small intestinal development. Specific tissue fixation preserves αSMA arrangement in low αSMA expressing cells that are conventionally undetectable under αSMA immunofluorescent stain due to inappropriate fixative-caused artificial actin depolymerization. Parallel analysis of αSMA carbonylation allows estimation of oxidative damage in gut muscular lineage. This approach improves the molecular specificity offered by commercialized kits that estimate total protein carbonyl level in cell lysates without protein specificity.

For complete details on the use and execution of this protocol, please refer to Hu et al. (2021).

## Highlights

• Optimal fixative improves αSMA immunofluorescent staining in the mouse intestine
• Improved cellular resolution of αSMA protein in whole mount immunofluorescent staining
• Semiquantitative measurement of oxidative damage on specific tissue proteins *in vivo*
• This protocol identifies the size distribution of total protein carbonyl in animal tissue

## Abstract (editor highlights)

Publisher’s note: Undertaking any experimental protocol requires adherence to local institutional guidelines for laboratory safety and ethics.

## Graphical abstract

**Figure:**
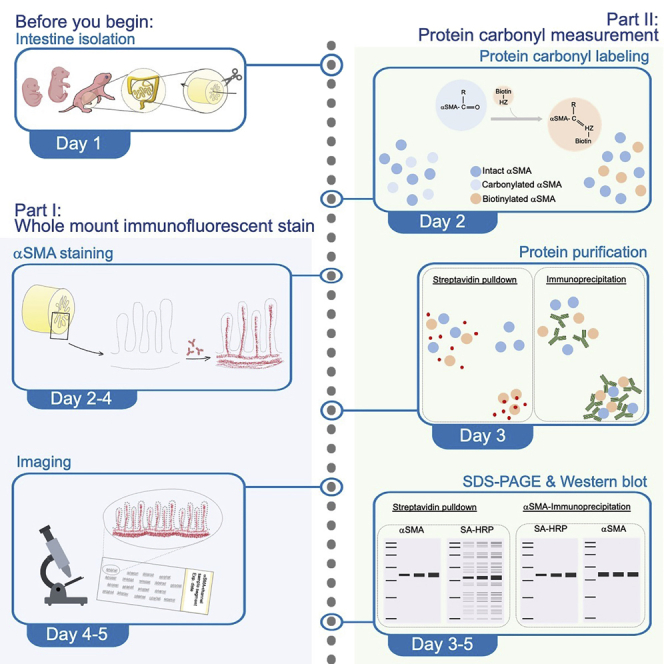

## Before you begin

### Institutional permissions

All experiments adhered to guidelines of the Institutional Animal Care and Use Committee of Cornell University, under the Animal Welfare Assurance on file with the Office of Laboratory Animal Welfare.

Permissions for animal experiment from the relevant institutions. Any experiments on live vertebrates or higher invertebrates must be performed in accordance with relevant institutional and national guidelines and regulations. Permissions for animal experiments from the relevant institutions are required for the following experiments.

***Note:*** The whole mount immunofluorescent stain protocol works well in mouse embryonic day (E) 16.5–18.5 and neonatal postnatal day (P) 0–9 small intestine. Please refer to previously published protocol (Bernier-Latmani and Petrova, 2016; Suh et al., 2018) for whole mount immunofluorescent stain in adult mouse intestine.

### Intestine isolation

**Timing: 1 h**

**Figure 1:**
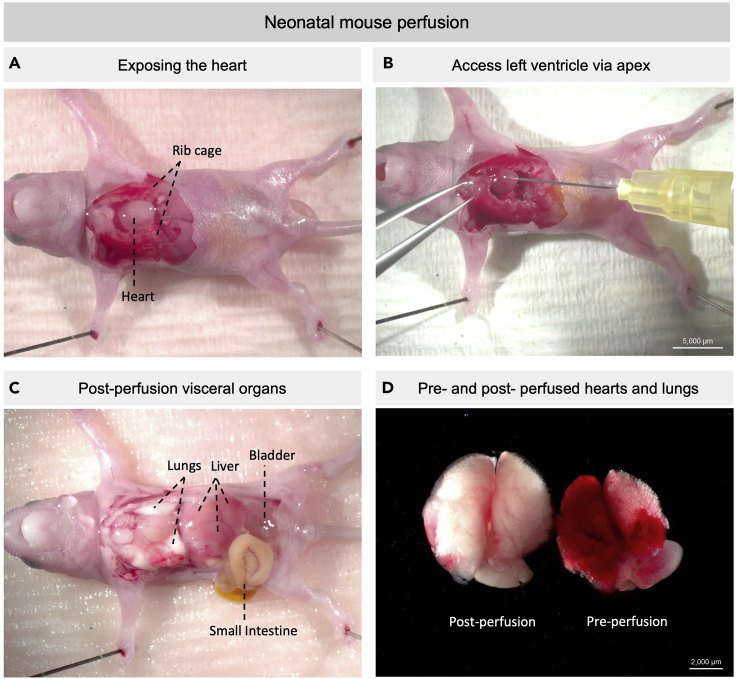
Neonatal mouse cardiac perfusion (A) Representative image of a P1.5 neonatal mouse pinned on a Styrofoam dissection tray. (B) Use a 31 G syringe needle to inject 10 mL of ice-cold PBS into the left ventricle. Access the left ventricle via the apex. (C) Cut open the abdomen wall to access the peritoneal cavity. Visceral organs should turn pale after a successful perfusion. Otherwise, more PBS injection is recommended. (D) Comparison of two pairs of heart and lungs. Left: heart and lungs isolated from a PBS perfused P1.5 mouse. Right: heart and lungs isolated from a P1.5 mouse without perfusion. (A–C) Scale bar = 5,000 μm. (D) Scale bar = 2,000 μm.

**Figure 2:**
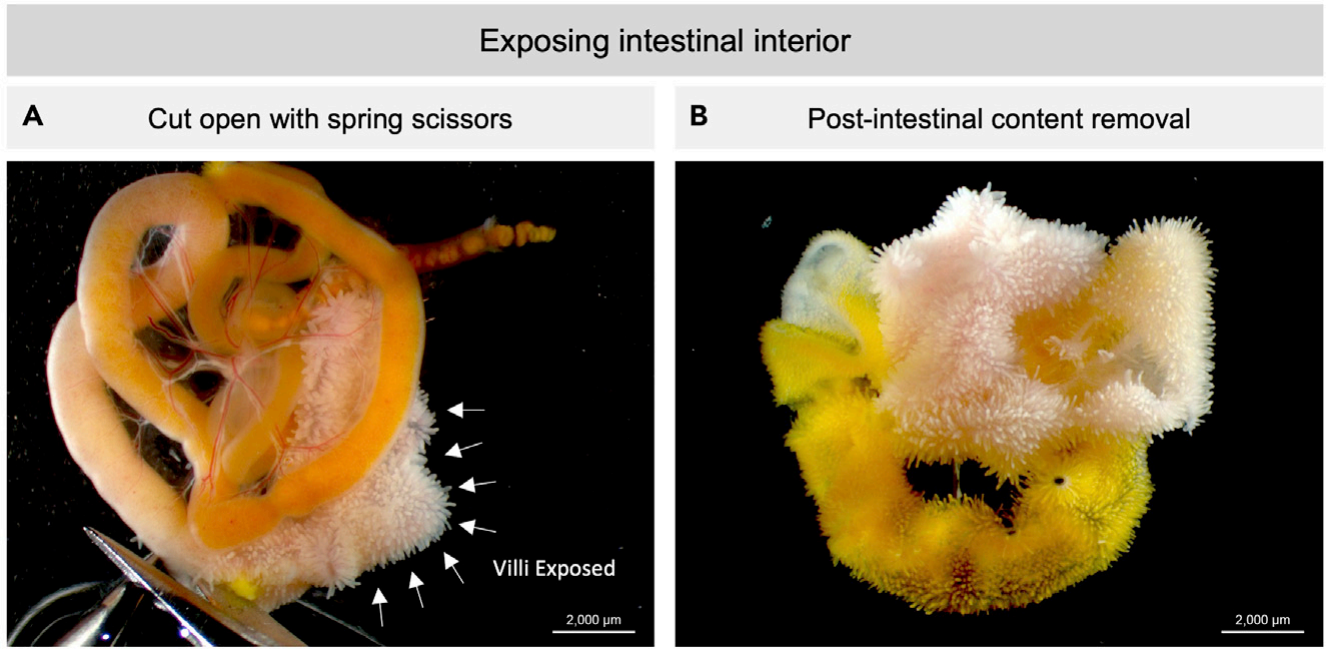
Exposing intestinal interior (A) Representative image of a P1.5 neonatal mouse small intestine. A pair of spring scissors was used to cut open the small intestine. A pair of fine forceps to support and move the tissue is recommended. White arrows indicate villi exposed. (B) Complete exposure of intestinal interior from (A) with intestinal content removed. (A and B) Scale bar = 2,000 μm.

1. Tissue isolation.
  a. If collecting samples from embryonic tissues, euthanize the pregnant dam before embryo isolation per institutional IACUC (Institutional Animal Care and Use Committee) protocol.
    **Recommended**: To reduce auto-fluorescent background from blood cells, proceed to step 2 for an alternative dissection protocol with PBS perfusion.
  b. Decapitate embryos or neonates before whole gut isolation.
  c. Pin down the paws of the mouse on a Styrofoam dissection tray with the ventral side upward (Figure 1).
  d. Dissect out the whole intestine.
    i. Cut open the skin and peritoneum to access and expose the intestine from the ventral side of abdominal cavity.
      ***Optional:*** Open the rib cage and remove all visceral organs from the thymus to the colon in younger mice to avoid mechanically injuring the digestive system.
    ii. Use fine forceps to lift visceral organs from the body cavity while using spring scissors to detach the connective tissues on the dorsal side.
    iii. Place intestine in ice-cold PBS and remove surrounding unwanted tissues.
    iv. Expose the intestinal interior by inserting spring scissors into the duodenal end of the intestine and gently begin snipping along the length of the intestine (Figure 2). Remove intestinal contents by shaking the intestines with forceps in ice-cold PBS several times.
    v. If proceeding to immunofluorescent staining, follow instructions in step 3. If proceeding to protein carbonyl detection, skip step 3 and follow instructions in step 4.
2. Tissue isolation (alternative tissue dissection with embryonic and neonatal heart perfusion).
  a. Euthanize the embryos or neonates by hypothermia instead of decapitation. Keep the mice on a weighing boat on ice for 7–10 min until there is no body movement.
  b. Pin down the paws of the mouse on a Styrofoam dissection tray with the ventral side upward.
  c. Create a minimal wound at the lower edge of rib cage and expose the heart apex (Figure 2A).
  d. Steadily and slowly inject 10 mL of ice-cold PBS into the left ventricle through apex with 31G × ½″ needle (Figure 2B). The organs (lungs, liver, mesentery) will turn pale if the perfusion is successful (Figures 2C and 2D).
  e. Proceed to step 1c.

**CRITICAL:** Do not leave the carcass on ice for too long or coagulation will interfere the perfusion efficiency. Create minimal wound while accessing the heart, as a bigger lesion opens up the circulation and leads to inefficient perfusion. If a tail snip was taken before the perfusion, perfusion fluid will come out from the wound created. The color of perfusion fluid will become transparent after most blood cells are perfused out of the circulation after 5–10 mL of PBS perfusion. Perfusion efficiency in neonatal mice is less efficient than in adults as the closure of Foramen Ovale is incomplete (Cole-Jeffrey et al., 2012).

**Figure 3:**
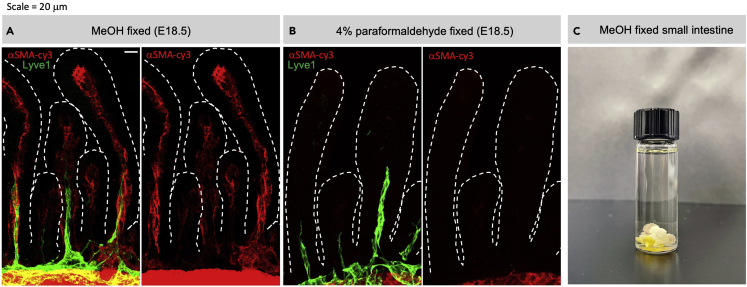
Methanol fixed small intestine (A) Villous αSMA (red) and lymphatic endothelial cell marker Lyve1 (green) co-labeling in a MeOH fixed E18.5 intestine. (B) Villous αSMA (red) and lymphatic endothelial cell marker Lyve1 (green) co-labeling in a 4% paraformaldehyde fixed E18.5 intestine. (C) Representative image of a P1.5 mouse small intestine fixed in ice-cold methanol. Tissue should dehydrate and sink to the bottom immediately. (A and B) Scale bar = 20 μm.

**CRITICAL:** Some perfusion protocols perfuse the carcass with formaldehyde-based fixatives. However, this depolymerizes αSMA cellular arrangement in cells with weak αSMA expression (Alarcon-Martinez et al., 2018). Avoid formaldehyde-based fixatives before immunofluorescent staining is done. Formaldehyde-based fixatives significantly decrease the αSMA signal (Figures 3A and 3B).

3. Tissue fixation for immunofluorescent staining.
  a. Place tissue of interest into ice-cold 100% methanol. Agitate gently to flatten the tissue and keep on ice to equilibrate. The yellow color of bile-derived pigments should be dissolved into the methanol. Change into fresh 100% ice-cold methanol every 5 min for 2–3 times until the fixation buffer is colorless.
    **CRITICAL:** Successfully fixed dehydrated intestine will sink to the bottom of the tube immediately (Figure 3C).
  b. Store the tissue in fresh 100% ice-cold methanol at −20°C until ready to use.
    **Pause point:** Tissues can be stored at −20°C for at least three months.
    **CRITICAL:** It is necessary to expose the interior of the gut tube prior to fixation if the villous structure is of interest. The villous structure will not be preserved if sample is immersed in methanol without exposing the interior and removing the intestinal content. Methanol fixation can be adapted for fixing other organs (Alarcon-Martinez et al., 2018).
    **CRITICAL:** If 3D structure is not of primary interest. Methanol fixed tissues can be prepared for cryoembedding from this point.

4. Flash freeze tissue in liquid nitrogen for protein carbonyl measurement. Store tissues at −80°C until use.

**Pause point:** Tissues can be stored at −80°C for at least three months.

**CRITICAL:** For embryonic and neonatal tissues where genotyping ahead of time is not possible, collect a piece of tissue such as tail or limb for genotyping before isolating the gut. Tissues can be stored at −20°C (methanol fixed) or −80°C (flash frozen) for at least three months.

## Key resources table

**Table:** 

REAGENT or RESOURCE	SOURCE	IDENTIFIER
**Antibodies**		

Anti-Actin, α-Smooth Muscle (1:400 dilution for western blotting)	Sigma-Aldrich	A2547
Anti-Actin, α-Smooth Muscle-FITC (1:100 dilution for whole mount immunofluorescent stain)	Sigma-Aldrich	F3777
Anti-Actin, α-Smooth Muscle–Cy3™ (1:100 dilution for whole mount immunofluorescent stain)	Sigma-Aldrich	C6198
Rabbit Anti-Mouse IgG H&L (HRP) (1:2000 dilution western blotting)	Abcam	ab6728
Alexa Fluor 488 goat anti-rabbit IgG (H+L) (1:500 dilution for whole mount immunofluorescent stain)	Fisher Scientific	A11070

**Chemicals, peptides, and recombinant proteins**		

Methanol ACS grade	PHARMCO-AAPER	339000ACS
Goat serum	Thermo Fisher Scientific	16210072
Bovine serum albumin	Sigma-Aldrich	A3059
Triton^TM^ X-100	Sigma-Aldrich	TX1568-1
Sodium Azide	Sigma-Aldrich	45-S2002
Sodium Acetate	Macron	7372-12
Sodium Chloride	Criterion	C7723
EDTA (0.5 M), pH 8.0 RNase-free	Thermo Fisher Scientific	AM9260G
Sodium dodecyl sulfate (SDS)	Avantor	97064-470
Tris Base	JT Baker	4109-06
Tween-20	Fisher Chemical	BP337500
Sodium Phosphate Dibasic Anhydrous	Fisher Chemical	S3743
Potassium Chloride	Macron	6858-06
Potassium Phosphate Monobasic Crystal	Macron	7100-12
DAPI	Thermo Fisher Scientific	D1306
Prolong Gold antifade reagent 10 mL without DAPI	Invitrogen	P36930
FocusClear™	Celexplorer	FC-101
EZ-Link™ Hydrazide-Biotin	Thermo Scientific	21339
Peroxidase Streptavidin	Jackson ImmunoResearch	016030084
Pierce™ High Capacity Streptavidin Agarose	Thermo Scientific	20357
Protein G Sepharose beads	Abcam	ab193259
Western Lightning Plus-ECL	PerkinElmer Inc.	NEL104001EA
Halt™ Protease and Phosphatase Inhibitor Cocktail	Thermo Fisher Scientific	78336
Pierce™ DTT (Dithiothreitol), No-Weigh™ Format	Thermo Fisher Scientific	A39255
Laemmli 2× Concentrate	Sigma-Aldrich	S3401-10VL
10× Tris/Glycine/SDS buffer	Bio-Rad	161-0732
10× Tris/Glycine Buffer	Bio-Rad	161-0734
8%–16% Criterion™ TGX Stain-Free™ Protein Gel	Bio-Rad	5678104
Precision Plus Protein Dual Color Standards	Bio-Rad	1610374
Dimethyl Sulfoxide (DMSO)	Sigma-Aldrich	D2650
Quick Start™ Bradford 1× Dye Reagent	Bio-Rad	5000205
Ponceau S Staining Solution	Cell Signaling Technology	59803

**Software and algorithms**		

FIJI	National Institutes of Health	http://fiji.sc; RRID: SCR_002285
Imaris 9.5	Bitplane	https://imaris.oxinst.com/; RRID: SCR_007370

**Biological samples**		

Male or female mouse intestine	Wildtype; Pitx2^ASE/+^ ; Pitx2^ASE/ASE^	Embryonic day 16.5 Embryonic day 18.5 Postnatal day 1.5 Postnatal day 9
Others		
Dissection microscope	Zeiss	SteREO Discovery. V12
Fine scissors	Fine Science Tools	14558-09
Insect pins #3	United Scientific	IPIN03-PK100
Styrofoam dissection tray	Foam fabricators, ltd.	470149-646
Fine forceps	Fine Science Tools	11254-20 or style #5
Spring scissors	Fine Science Tools	91501-09, 15000-04
Petri Dishes (100 × 15 mm)	VWR®	25384-342
TSK STERiJECT® Premium Needles 31G × 13 mm	Air-Tite Products Co., Inc.	TSK3113
Syringe needles25G × 1½″	BD	305127
1 and 5 mL syringe	BD	309628, 309646
Confocal microscope for acquiring tissue slice 3D-images with filters capable of capturing FTIC and/or Cy3 signal	Zeiss	LSM880
Shaker	Reliable Scientific Inc	55D 11 × 14
Orbital shaker	Boekel Scientific	260100
Microscope slides	Fisherband^TM^	12-550-15
Cover glass, 24 × 60 mm	Laboratory Products Sales Inc.	M141910
1 mL syringe and 0.2-micron syringe filter units	VWR®	28145-477
Tissue grinder and pestle	BioSpec Products	SpiralPestle™ And MicroTube Homogenizer 1017MC tissue grinding kit or equivalent
Centrifuge compatible for 150–18,000 g at 4°C	eppendorf	5430
Heat block compatible for 98°C	VWR®	75838-282
Amicon Ultra 0.5 mL DNA/Protein centrifugal filters or equivalent	Millipore	UFC501024
SDS-PAGE gel running and blot transferring systems, and a power supply	Bio-Rad	Criterion^TM^ Cell, Criterion^TM^ Blotter, PowerPac^TM^ Basic
Imager for visualizing chemiluminescent western blotting substrates	Bio-Rad	ChemiDoc^TM^ MP Imaging System 2011 model

## Materials and equipment

**Figure 4:**
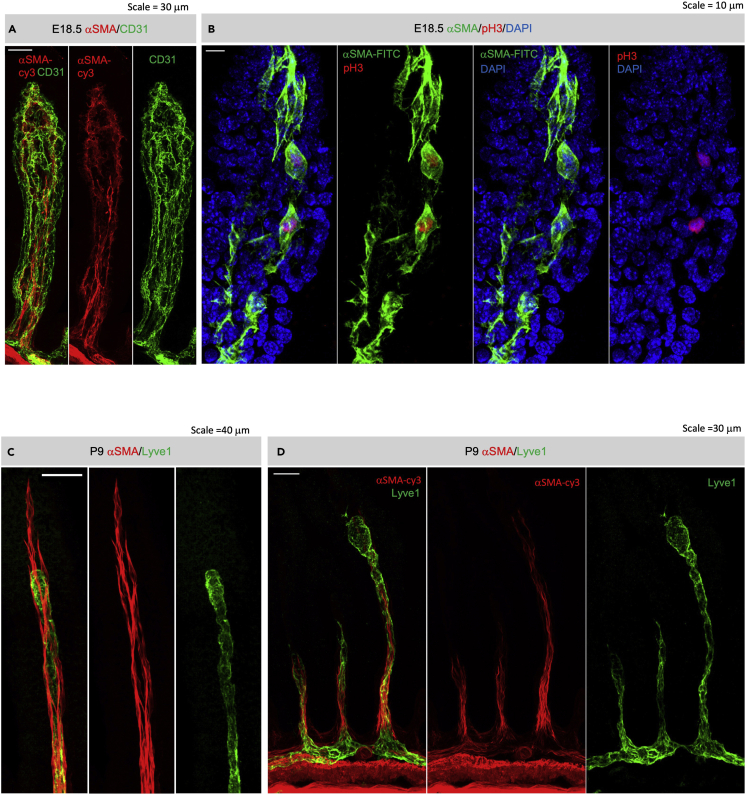
Whole mount immunofluorescent staining of αSMA in villous stroma (A) Co-labeling of αSMA (red) and pan-endothelial cell marker CD31 (green) in E18.5 jejunum villus. (B) Co-labeling of αSMA (green) and phospho S10 histone H3 (pH3, red) in E18.5 jejunum villous. (C and D) Co-labeling of αSMA (red) and lymphatic endothelial cell marker Lyve1 (green) in P9 jejunum villi. Note the structure of vascular smooth muscle cells in the *lamina propria*, and the circular and longitudinal muscles in the *muscularis externa* in (D). (A and D) Scale bar = 30 μm; (B) Scale bar = 10 μm; (C) Scale bar = 40 μm.

***Alternatives:*** We have successfully used the following antibodies as co-staining markers along with αSMA: vascular endothelial cell marker CD31/PECAM-1 (BD Sciences, 553370) (Figure 4A), cell proliferation marker phospho S10 of Histone H3 (Abcam, Ab5176) (Figure 4B), and lymphatic endothelial cell marker Lyve1 (Abcam, Ab14917) (Figures 4C and 4D). These antibodies can be replaced by other antibodies of interest, but the compatibility of such antibodies against methanol fixed antigens needs to be tested beforehand.

***Alternatives:*** This protocol uses the Criterion^TM^ cell, blotter, and precast gels for SDS-PAGE. We visualized our proteins with chemiluminescent substrates and a Bio-Rad ChemiDoc MP system. Other equivalent settings should serve the same purpose.

**CRITICAL:** H_2_O in this protocol should be high quality water such as double distilled, RO or milliQ equivalent.

**Table:** 10× PBS

Reagent	Final concentration	Amount
Sodium Chloride (NaCl)	1.37 M	80 g
Potassium Chloride (KCl)	27 mM	2 g
Sodium Phosphate Dibasic Anhydrous	100 mM	14.4 g
Potassium Phosphate Monobasic Crystal	18 mM	2.4 g
H_2_O	n/a	Top to 1 L
**Total**	**n/a**	**1 L**

Adjust the pH to 6.8 before bringing the final volume to 1 L. 10× PBS can be stored at 20°C–25°C for months as long as the pH is still 6.8.

**Table:** Whole Mount Immunofluorescent Blocking reagent

Reagent	Final concentration	Amount
Heat inactivated goat serum	5%	2.5 mL
Bovine serum albumin	0.5%	0.25 g
Triton-X 100	0.3%	0.15 mL
Sodium azide	0.1%	0.05 g
1× PBS	n/a	Top to 50 mL
**Total**	**n/a**	**50 mL**

Store at 4°C for a week, or aliquot and store at −20°C for months.

**CRITICAL:** Sodium azide is a hazardous reagent, use a chemical fume hood and wear protective gloves and mask when handling the chemical.

***Alternatives:*** Goat serum can be replaced by donkey serum as described previously (Bernier-Latmani and Petrova, 2016).

**Table:** Whole Mount Immunofluorescent Wash buffer (PBST)

Reagent	Final concentration	Amount
Triton-X 100	0.3%	1.5 mL
PBS	n/a	500 mL
**Total**	**n/a**	**500 mL**

Can be stored at 20°C–25°C for months.

**Table:** Biotin-Hydrazide homogenization buffer, pH = 5.5

Reagent	Final concentration	Amount
Sodium Acetate	100 mM	8.203 g
Sodium Chloride	20 mM	1.17 g
EDTA	0.1 mM	0.2 mL from 500 mM stock
H_2_O	n/a	Top to 1 L
**Total**	**n/a**	**1 L**

Adjust pH to 5.5 before bringing the final volume to 1 L. This buffer can be stored at 20°C–25°C for months.

**Table:** 20% SDS stock solution

Reagent	Final concentration	Amount
Biotin-Hydrazide homogenization buffer, pH = 5.5	n/a	4 mL
Halt™ Protease and Phosphatase Inhibitor Cocktail	1×	40 μL
SDS	20%	0.8 g
**Total**	**n/a**	**4 mL**

Make fresh before use.

**Table:** SDS-PAGE electrophoresis running buffer

Reagent	Final concentration	Amount
10× Tris/Glycine/SDS buffer	1×	100 mL
H_2_O	n/a	900 mL
**Total**	**n/a**	**1 L**

Can be stored at 20°C–25°C for months.

***Note:*** 10× premixed Tris/Glycine/SDS buffer contains 25 mM Tris, 192 mM glycine, 0.1% SDS, pH 8.3.

**Table:** SDS-PAGE transfer buffer

Reagent	Final concentration	Amount
10× Tris/Glycine buffer	1×	100 mL
Methanol	20%	200 mL
H_2_O	n/a	700 mL
**Total**	**n/a**	**1 L**

Store at 4°C for immediate use. Can be reused up to three times.

***Note:*** 10× premixed Tris/Glycine buffer contains 25 mM Tris, 192 mM glycine, pH 8.3.

**CRITICAL:** Methanol is a hazardous reagent, use a chemical fume hood and wear protective gloves and mask when handling the chemical.

**Table:** 10× TBS (pH = 7.6)

Reagent	Final concentration	Amount
Tris	200 mM	24 g
Sodium Chloride	1500 mM	88 g
H_2_O	n/a	Top to 1 L
**Total**	**n/a**	**1 L**

Adjust the pH to 7.6 before bringing the final volume to 1 L. 10× TBS can be stored at 20°C–25°C for months.

**Table:** Western blot wash buffer (TBST)

Reagent	Final concentration	Amount
Tween 20	0.05%	500 μL
10× TBS	1×	100 mL
H_2_O	n/a	900 mL
**Total**	**n/a**	**1 L**

Can be stored at 20°C–25°C for months.

## Step-by-step method details

### Part I: Whole mount immunofluorescent detection of αSMA

**Timing: 2–3 days**

αSMA proteins are labeled by fluorescent conjugated antibodies in mouse intestine tissue slices in this part.

***Note:*** The following protocol is an optimization of a previously published protocol in adult mouse intestine (Bernier-Latmani and Petrova, 2016; Suh et al., 2018). The following protocol is recommended for younger tissues that are less mature and have better tissue permeability than older tissues. Representative images of the whole mount immunofluorescent stain are available in Figure 4.

***Note:*** Conjugated αSMA clone 1A4 antibodies are recommended when staining mouse tissue to minimize background signal.

***Note:*** Methanol fixation will quench intrinsic fluorescent signal such as transgenic GFP or tdTomato. However, using anti-GFP or anti-RFP antibodies, respectively, to detect quenched fluorescent proteins can circumvent this inconvenience.

1. Use spring scissors to separate tissue of interest from methanol-fixed samples into smaller pieces for better penetration. Collect into a round end 2 mL Eppendorf tube in PBS.
2. Wash the tissue 3 times with ice-cold PBS for 5 min each.

***Optional:*** If working with intestines collected from mice older than P9: Before transferring to blocking reagent, permeabilize tissues in PBST-100 (0.3% Triton-X-100) for 6 h at 4°C with gentle rocking. Block for 3 h at 4°C with mild agitation. Use enough blocking reagent to completely submerge all tissues to avoid tissue drying.

3. Incubate with anti-αSMA antibody and other primary antibodies of interest at 4°C for 12–16 h with gentle agitation. Primary antibodies are diluted in fresh blocking reagent with optimal dilution titers. We use 1:100-1:200 for conjugated αSMA antibody.

***Note:*** The antibody-blocking solution should be filtered with 0.22 μm filter before adding to the samples. Avoid light when handling conjugated antibodies.

**Pause point:** Samples can be left in primary antibodies at 4°C for more than 1 day if needed.

4. (Go to step 7 if using conjugated antibodies and do not need secondary antibody incubation). Wash with ice-cold PBST-100 (0.3% Triton-100) with gentle rocking at 4°C for five hours, change buffer every hour for 5 times.

***Note:*** Some primary antibody solutions can be reused if stored at −20°C. However, we do not recommend reusing antibodies that cannot be refrozen after thawing.

***Note:*** Fluorescent dyes are light sensitive; minimize ambient light exposure when handling fluorescent dye-conjugated antibodies.

5. Incubate with secondary antibody at 4°C with gentle agitation for 12–16 h.

***Optional:*** Can incubate tissues with DAPI (1:1000 dilution in secondary antibody-blocking solution) to visualize cell nuclei if needed.

***Note:*** Avoid prolonged secondary antibody incubation to minimize background caused by non-specific secondary antibody binding.

***Note:*** We recommend filtering the secondary antibody with 0.22 μm filter before applying to the tissues.

6. Wash with ice-cold PBST-100 (0.3% Triton-X-100) every 30 min, 10 times, at 4°C with mild agitation.
7. Wash with ice-cold PBS for 10 min, 3 times, at 4°C with mild agitation.
8. Fix with 4% PFA/PBS at 4°C for 12–16 h.

***Note:*** For tissues thicker than 0.5 cm, we recommend leaving at 4% PFA for two days.

9. Wash with ice-cold PBS for 10 min, 3 times.

***Note:*** For tissue embedding, slice the intestine with spring scissors and mount in FocusClear^TM^/Prolong Gold antifade reagent for confocal imaging (Figure 4) (Bernier-Latmani and Petrova, 2016). The final thickness of the embedded tissue should be no thicker than 2–3 layers of villi.

**Figure 5:**
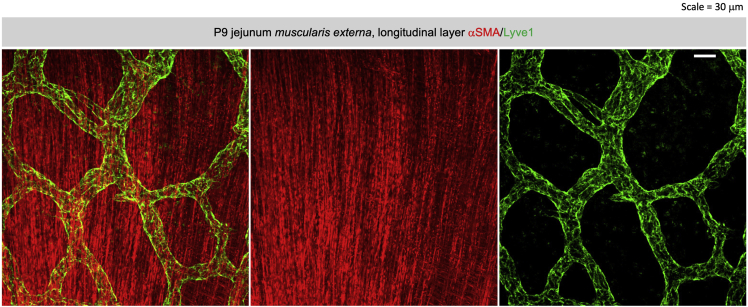
Whole mount immunofluorescent staining of αSMA *in muscularis externa* Co-labeling of αSMA (red) and Lyve1 (green) in P9 jejunum muscularis externa, note the arrangement of longitudinal muscles. Scale bar = 30 μm.

***Note:*** If the goal is to visualize details in *muscularis externa*, we recommend cutting the intestine into pieces and mount with villi facing downward (Figure 5). Alternatively, tissues can be embedded and sectioned after whole mount immunofluorescent staining if needed.

***Note:*** Please refer to (Bernier-Latmani and Petrova, 2016; Suh et al., 2018) for detailed tissue embedding and confocal imaging instructions.

### Part II: Tissue protein carbonyl measurement

#### Protein extraction

**Timing: 3 h**

Extract total proteins from the intestine for downstream analysis. This is a separate protocol parallel to **α**SMA immunofluorescent detection described in Part I.

**Figure 6:**
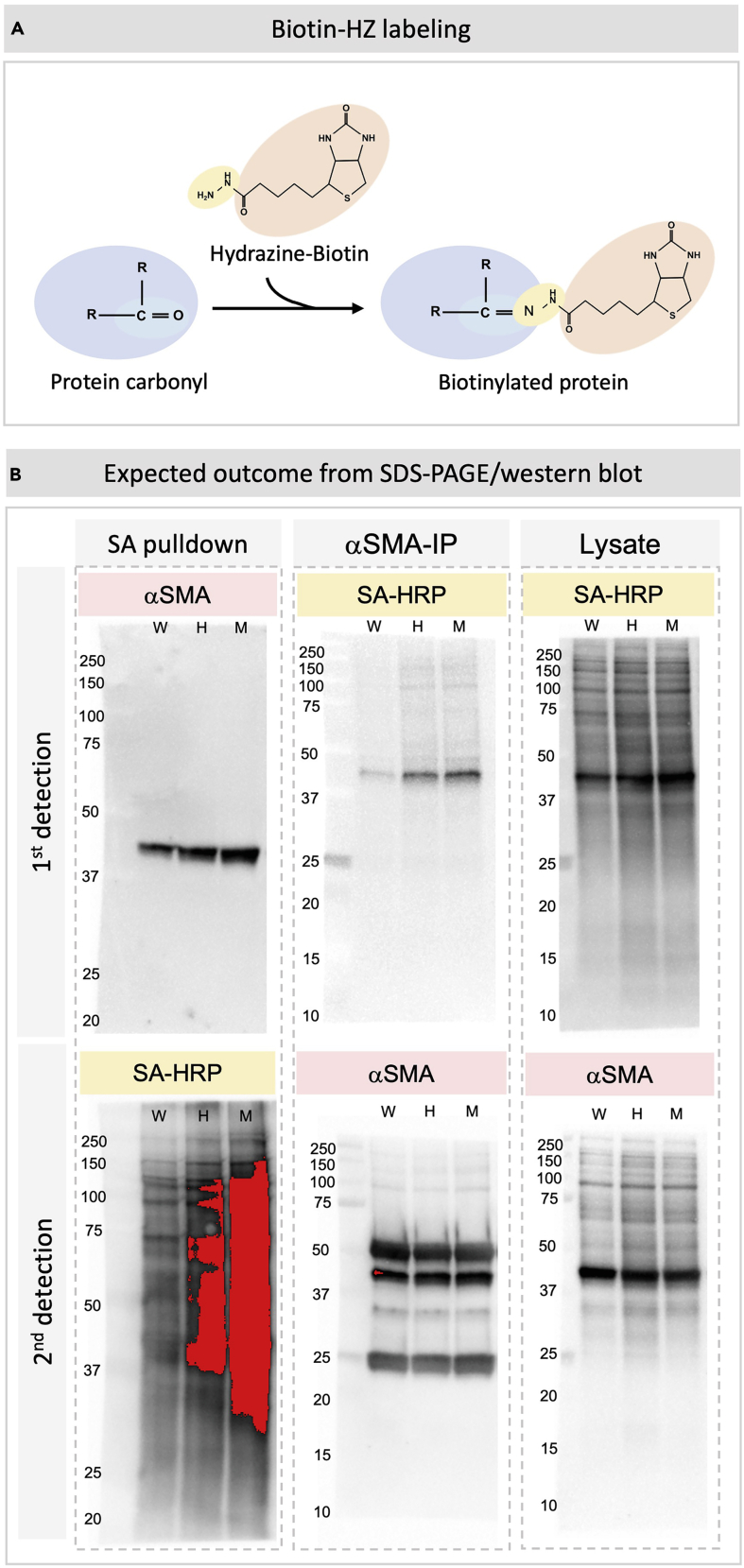
Detection of protein carbonyls (A) The biotin conjugated hydrazide reagent reacts with carbonyl group on oxidized proteins and biotinylates carbonylated proteins. (B) Representative outcome from Part II. Three samples (WT, wildtype; HET, heterozygote; and MUT, homozygotic mutant) collected from P1.5 *Pitx2*^*ASE*^ littermates are measured in this experiment. Samples were processed as described in Part II and blotted on three separated membranes (Left: samples purified with streptavidin (SA) pulldown assay, middle: samples purified with αSMA-immunoprecipitation, right: lysates without further purification/enrichment). Note that the same 3 blots are first probed in primary detection (shown on top), then probed in secondary detection (shown in bottom) on the next day. Reagents (red: αSMA antibody, yellow: streptavidin-HRP) used for protein detection in both primary and secondary detections are color-labeled. The red color indicates saturated signal on the blot.

***Note:*** Keep the tubes on ice at all times to avoid protein degradation.

***Note:*** Carbonyl derivatives (protein-C=O; Figure 6A) are irreversible products of ROS-mediated protein oxidation. Protein carbonyl groups are often used as a general biomarker for oxidative injury (Fedorova et al., 2014; Dalle-Donne et al., 2003; Chevion et al., 2000), and we use protein carbonyl as a marker to quantify oxidative injury. There will always be baseline protein carbonyls in the cell, therefore it is essential to include a control sample versus the experimental group.

10. Homogenize tissue samples.
  a. Retrieve and thaw samples from −80°C on ice for 5–10 min.
  b. Make homogenization buffer by adding phosphatase/protease inhibitor cocktail to Biotin-Hydrazide homogenization buffer per label description. Add an adequate amount of buffer to the samples. We recommend adding 500 mL to each intestine sample taken from E18.5-P1.5 mouse. Larger tissue may need more buffer.
  c. Use a tissue grinder pestle to homogenize tissues completely until no visible tissue chunks remain.
  d. Shear genomic DNA by passing the homogenized samples up and down through a 1 mL syringe with a needle <25 G. Do this 30 times on ice.

11. Spin at 150 g for 10 min at 4°C.
12. Collect supernatant into a new tube and add SDS to make final concentration of 2%.

***Note:*** We recommend making 20% SDS stock solution in homogenization buffer with protease inhibitor beforehand. Add 1:10 v/v of the premade SDS stock solution to the collected supernatant to make final SDS concentration of 2% in the sample tubes.

13. Heat the sample with SDS at 65°C for 5 min.
14. Spin at 18,000 g for 1 h at 4°C.
15. Collect the supernatant into a new tube and measure protein concentration for each sample.

***Note:*** SDS interferes with several protein assays. Most commercial protein assays include literature on detergent compatibility, which should be consulted prior to use. Protein concentration can be measured at this step with a protein assay compatible with 2% SDS, if samples are run undiluted. To circumvent SDS interference, samples can be diluted to a level of SDS within the protein assay compatibility range. Alternatively, the protein concentration can be determined prior to SDS addition (step 12), and the final concentration calculation adjusted for the volume of SDS added. For any protein assay, a buffer only control should be run to assess any signal contributed by buffer components.

#### Biotin-hydrazide incubation

**Timing: 4 h**

This step labels protein carbonyl with biotin-hydrazide.

16. Take 100–200 μg of protein samples from each tube, top to 400 μL with homogenization buffer with protease inhibitor from step 12.
17. Add 10 μL of EZ-link Hydrazide-biotin (stock solution: 50 mM in DMSO, store at −20°C) to the sample, bringing the total volume of labeling mix to 410 μL.
18. Incubate the labeling mix at 20°C–25°C with gentle agitation for 2 h.
19. Concentrate sample and remove unreacted Hydrazide-biotin and SDS.
  a. Add samples (410 μL) to an Amicon Ultra-0.5 mL centrifugal filter, prepped following manufacturer’s instructions.
  b. Spin at 14,000 g for 10 min.
  c. Discard flowthrough, add 400 μL PBS, then spin at 14,000 g for 10 min. Repeat twice.
  d. Recover the samples into a clean tube by spinning at 1,000 g for 2 min. The volume of concentrated samples should be around 40 μL. Add another 20 μL PBS to bring sample volume to 60 μL. Measure protein concentration again.

***Alternatives:*** We use Amicon Ultra 0.5 mL DNA/Protein centrifugal filters, UFC501024, Millipore for step 19. Other protein desalting and concentrating columns or methods such as TCA precipitation should be equally effective.

***Note:*** Concentrated samples should be arranged into three aliquots for streptavidin pulldown assay, αSMA immunoprecipitation (IP), and lysate control. We recommend using roughly 45% of the protein samples for Streptavidin pulldown, 45% for αSMA IP assay, and 10% for the lysate control (step 31).

#### Streptavidin pulldown

**Timing: 1.5 days**

This step isolates all Biotin-Hydrazide labeled proteins (total carbonylated proteins) in the lysate.

20. Pre-wash Streptavidin beads and equilibrate in PBS.
  a. Mix beads gently by pipetting or inversion of bottle until a homogeneous suspension. Use a cut-off pipette tip to transfer 100 μL of resuspend streptavidin beads to a clean 1.7 mL tube.
    ***Note:*** Do not vortex the beads.
  b. Spin at 500 g for 1 min at 20°C–25°C. Discard supernatant.
  c. Resuspend the beads with 500 μL of PBS (10 volumes of PBS to beads), centrifuge at 500 g for 2 min and discard supernatant. Repeat two more times.
  d. Resuspend the beads in 50 μL of PBS.
  e. Aliquot beads into clean tubes.
21. Add an aliquot of protein samples from step 19 to the tubes with streptavidin beads.

***Note:*** We use approximately 15 μL of resuspended beads from step 20 for 75–100 μg of biotinylated proteins from step 19, making a 50% beads-sample slurry for step 21.

22. Gently mix the samples and streptavidin beads. Keep rocking for 12–16 h at 4°C.
23. Remove unbound proteins.
  a. Spin down the beads at 2,000 g for 2 min in 20°C–25°C. Collect supernatant to confirm pulldown efficiency later (see step 32).
  b. Resuspend beads with 1 mL of 0.01% SDS in PBS, centrifuge at 2,000 g for 2 min.
  c. Repeat step 23b four more times. Leave 30 μL of wash buffer in the last wash and resuspend the beads by gentle pipetting.
24. Protein elution.
  a. Add 30 μL of 2× SDS Laemmli sample buffer to the sample.
  b. Boil at 98°C on a heating block for 5 min.
  c. Centrifuge at 2,000 g for 2 min.
  d. Collect supernatant into a clean tube for SDS-PAGE. Avoid collecting beads.

**Pause point:** Samples can be frozen and kept at −80°C after step 24b for at least one week.

#### αSMA immunoprecipitation (IP)

**Timing: 1.5 days**

This step isolates all αSMA proteins (regardless of oxidative status) in the lysate.

25. (Start from step 19) Take 75–100 μg of protein samples and add 2–4 μL of unconjugated αSMA antibody clone 1A4. Incubate at 4°C with rotation for at least 12 h.

***Note:*** Leave for 12–16 h with step 22 if doing streptavidin pulldown in parallel.

***Note:*** The amount of αSMA antibody used for IP immunoprecipitation depends on αSMA abundance in the sample. Please scale up antibody volume if starting with more samples.

26. Pre-wash protein G Sepharose beads before use.
  a. Take 100 μL of protein G Sepharose beads suspension with a cut-off pipette tip, spin at 150 g at 4°C for 2 min. Discard supernatant.
  b. Wash with 1 mL PBS, spin down at 150 g at 4°C for 3 times.
  c. Aliquot protein G beads slurry to each tube, each tube should have 15–25 μL of protein G beads slurry. Make sure each tube gets equal amount of protein G beads.

***Note:*** Do not vortex the beads.

***Note:*** The αSMA antibody clone 1A4 is mouse IgG2a subtype, which would also be compatible with capture by protein A. If a different αSMA antibody is used for the IP, the choice of protein A and/or G beads for antibody capture should be based on the affinity of the antibody IgG subtype for protein A and G. This information is readily available from commercial supplier websites.

27. After 12–16 h incubation is complete (step 25), add biotin-hydrazide labeled protein-αSMA antibody mix to the tubes with protein G beads. The amount of sample mix to protein G beads slurry should be roughly 1:1 (v/v). Rotate at 4°C for 4 h.
28. Spin at 150 g at 4°C for 2 min to collect beads. Save 20 μL supernatant into a separate tube for IP efficiency control. Remove and discard remaining supernatant, containing unbound material.
29. Wash beads with 500 μL PBST-100 (1% Triton-X-100), spin at 150 g at 4°C for 5 times.
30. Elution:
  a. Add 30 μL of 2× Laemmli sample buffer with 100 mM DTT and boil on a heating block for 5 min.
  b. Spin at 150 g at 20°C–25°C for 5 min, collect supernatant for SDS-PAGE.

#### Preparing controls

**Timing: 30 min**

Control samples are processed in this step.

31. Lysate control (see step 19):
  a. Take 1/10 (g/g) of proteins used for pulldown/IP assay from dialyzed biotinylated samples.
  b. Bring the volume to 30 μL by adding PBS.
  c. Add 30 μL of 2× sample buffer.
  d. Boil on a heat block for 5 min, take 30 μL for SDS-PAGE.

***Note:*** The amount of sample mix to protein G beads should be at a minimum 1:1 (v/v).

32. Supernatant control for pulldown efficiency.
  a. (Start from step 23a and step 28) Take 5 μL of supernatants and add 25 μL of PBS.
  b. Add 30 μL of 2× sample buffer.
  c. Boil for 5 min, take 30 μL for SDS-PAGE.

#### SDS-PAGE and western blot

**Timing: 1.5 days**

Carbonylated proteins are separated and detected in this step.

33. Remove the gel comb and tape if using precast gels. Equilibrate the gel to 20°C–25°C.

***Optional:*** Researchers can also prepare their own polyacrylamide gels, versus using precast gels. Gel pouring systems (with instructions for preparation) are available from several suppliers (e.g., Bio-Rad min-PROTEAN handcast system).

34. Rinse/flush the wells thoroughly with running buffer before loading the samples.
35. Load samples and ladder accordingly.
36. Run the SDS-PAGE with 110 V for 90 min.
37. While the SDS-PAGE is running, prepare transfer buffer and cool at 4°C.
38. Soak nitrocellulose membrane in transfer buffer for 10 min and mark the orientation of membrane.
39. Complete a wet transfer at 60 V for 2 h.

***Note:*** This protocol uses the Criterion^TM^ cell, blotter, and precast 8%–16% gels for SDS-PAGE. Appropriate running time and voltage for SDS-PAGE and transfer should be adjusted for different percentage gels and/or transfer systems.

***Note:*** To compare the relative signal between samples, samples must be run and transferred on the same gel. Thus, all αSMA or all streptavidin pulldown samples should be run together on a single gel.

40. Make blocking solution during the wait: 5% BSA in TBST or 5% skim milk in TBST.

***Optional:*** Transfer efficiency and sample loading can be visualized with a Ponceau S staining solution. Wash membrane in TBST until the bands are no longer visible prior to continuing with step 41.

41. Incubate the blot in blocking reagent for 1 h at 20°C–25°C with gentle agitation.
42. Wash the membrane with TBST three times for 5 min each at 20°C–25°C.
43. Incubate in primary antibody (dilute in blocking reagent) or streptavidin-HRP (dilute in TBST) for 12–16 h at 4°C with gentle agitation.
  ***Note:*** In the initial detection, add the corresponding reagent to the blot:
  a. Streptavidin pulldown blot: αSMA Ab (detect carbonylated αSMA).
  b. αSMA-IP blot: Streptavidin-HRP (detect all carbonylated proteins in the purified αSMA samples).
  c. Lysate blot: αSMA Ab (detect total αSMA across samples, ideally band intensity at ∼42 kDa should be comparable between samples to demonstrate equal amount of αSMA expression across samples).
    ***Note:*** For blots incubated in streptavidin-HRP, skip to step 46.
    ***Note:*** Suggested antibody working concentrations are available in the manufacturer’s instructions, which may need to be adjusted for optimal signal. We used a 1:400 dilution for primary antibody (αSMA, A2547) in this step, followed with a 1:2000 dilution for secondary antibody (rabbit anti-mouse HRP, ab6728) in step 45. Blots were incubated with Streptavidin-HRP (016030084) at a concentration of 0.1 μg/mL.
    **Pause point:** Blot can be left in primary antibody for more than 16 h if needed.

44. Wash the blots with TBST three times for 5 min each at 20°C–25°C.
45. Incubate the streptavidin pulldown blot and lysate blot with secondary antibody (HRP conjugated anti-mouse secondary antibody in TBST or blocking reagent from 32) for 1 h at 20°C–25°C.
46. Wash the blots with TBST three times for 10 min each at 20°C–25°C.
47. Incubate the blots in fresh luminol buffer mix.

***Note:*** Many chemiluminescent western blotting substrates choices are available, and we used the PerkinElmer Western Lightning Plus-ECL substrate. We visualized our proteins with a Bio-Rad ChemiDoc MP system. Proteins can be visualized by exposing the blot to film or any comparable imager capable of chemiluminescence.

48. Incubate the blots in fresh luminol buffer mix for 1 h to saturate and deplete remaining HRP activity.
49. Return to step 42 for the secondary detection.
  ***Note:*** In the secondary detection, add the corresponding reagents to the blots for “primary Ab incubation”:
  a. Streptavidin pulldown blot: Streptavidin-HRP (for total carbonylated proteins comparison across samples).
  b. αSMA-IP blot: αSMA Ab (ideally band intensity ∼42 kDa should be comparable between samples to demonstrate comparable αSMA-IP efficiency across samples).
  c. Lysate blot: Streptavidin-HRP (for total carbonylated proteins comparison across samples, should show similar trend as in the Streptavidin pulldown blot).
    ***Note:*** The above-described protocol in Part II is an optimized version from previous protocol dealing with cultured cell lysate and crude adipose tissue extract (Grimsrud et al., 2007; Xu et al., 2014). The previous protocol did not involve specific protein purification steps we described in Streptavidin pulldown and αSMA immunoprecipitation.

## Expected outcomes

### Whole mount IF

**Figure 7:**
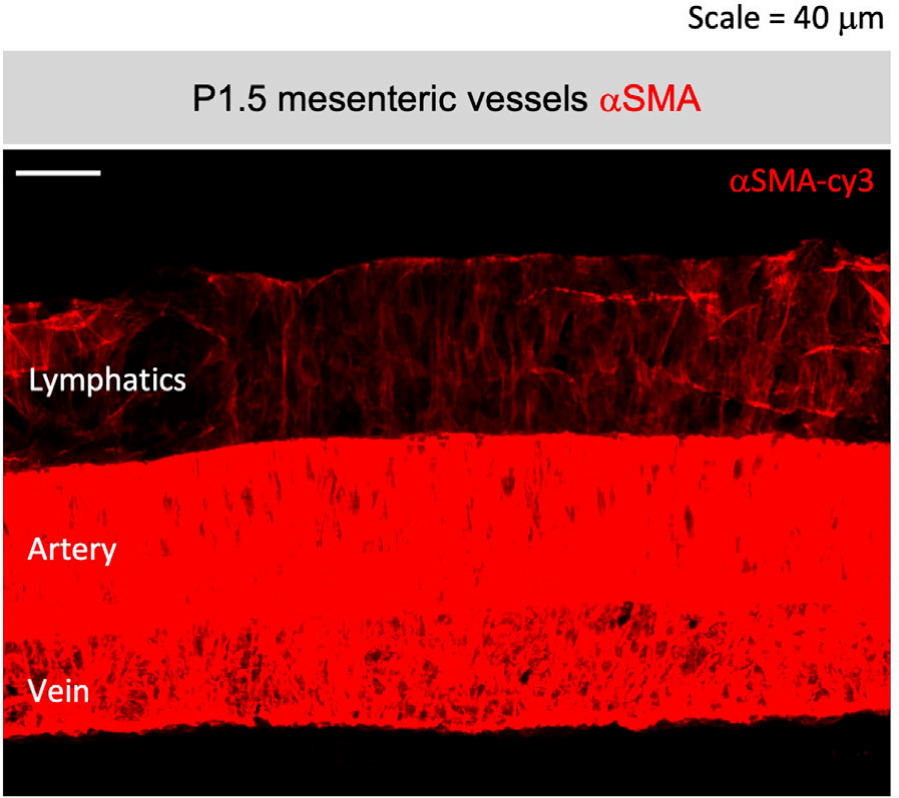
Whole mount immunofluorescent staining of αSMA in the dorsal mesentery αSMA (red) staining in P1.5 mesentery, note the arrangement vascular smooth muscle cells in the artery (middle), vein (bottom), and lymphatic collecting vessel (top). Scale bar = 40 μm.

Clear αSMA signal should be seen in both the *muscularis externa* and *lamina propria*. Vascular smooth muscle should also present in the submucosa (Figure 4D) and mesentery (Figure 7). The intensity of αSMA signal should be the strongest in the *muscularis externa*, followed by villous *lamina propria* vascular smooth muscle cells and villous axial smooth muscles, and the weakest in the villous blood-plexus associated smooth muscle “star cells.” The CD31-associated αSMA stain in the star cells is expected starting at E16.5. Axial smooth muscles can be seen no earlier than E17.5–18.5, with more axial muscle fibers established in older tissues. There might be different αSMA staining patterns in the lamina propria throughout the small intestine segments as the organ development in the anterior (duodenum) precedes the posterior (ileum) segments. It is important to note that variation in axial smooth muscle developmental timing was observed across different mouse genetic backgrounds (personal observation, data not shown).

### Carbonyl assay

A baseline level of protein carbonyl should be detected in all tissues regardless of genotypes. Sharp and clear bands instead of protein smears should present on the blot (Figure 6B). If the amount of streptavidin/ αSMA was saturating, there should be no detectable carbonylated proteins in the supernatants collected in step 23a, and no αSMA proteins in the supernatants collected from step 28. Quantitative recovery of material is important to reflect the total material in the samples and avoid erroneous conclusions based on differential protein recovery from the various genotypes. Visualization of the lysates (prior to pulldown) will establish effective biotin-hydrazide labeling as well as indicate the relative levels of carbonyl groups and actin across the samples. The presence of actin signal in the streptavidin pulldowns will be indicative of carbonylated (biotin-labeled) actin. Similarly, the presence of streptavidin signal in the actin IP samples will be representative of carbonylated (biotin-labeled) actin.

Expected experimental outcome from the primary detection:

Streptavidin pulldown blot (stain with αSMA ab): samples with more carbonylated αSMA should show a stronger band ∼42 kDa.

αSMA-IP blot (stain with streptavidin-HRP): samples with more carbonylated αSMA should show a stronger band ∼42 kDa.

Lysate blot (stain with αSMA ab): Bands of comparable intensity ∼42 kDa if αSMA expression remains comparable across samples.

Expected experimental outcome from the secondary detection:

Streptavidin pulldown blot (stain with streptavidin-HRP): samples with higher oxidative stress should have overall stronger bands. All carbonylated proteins will be detected in this step, specific target proteins being carbonylated will show enriched signal intensity at the corresponding molecular weight.

αSMA-IP blot (stain with αSMA ab): bands of comparable intensities ∼42 kDa if the expression and IP effectiveness were comparable across samples.

Lysate blot (stain with streptavidin-HRP): this blot should show the same trend as in the streptavidin pulldown blot.

## Limitations

### Whole mount αSMA IF

This protocol permits detection of trace αSMA protein detection by preserving the depolymerization-prone antigens with methanol fixation. Nevertheless, methanol fixation may impede the detection of certain antigens that require formaldehyde based-fixation. Some downstream assays are not compatible with methanol fixed tissues as well. For example, formaldehyde-based fixation is recommended in TUNEL assay for minimal DNA breakage. For histological details, we recommend doing Formalin-Fixed-Paraffin-Embedded (FFPE) procedure for H&E stain in parallel to this protocol.

### Carbonyl assay

Protein carbonyl is an irreversible protein oxidation product generated under extreme oxidative stress. The stability of protein carbonyl makes it a cumulated injury marker, while providing limited information on the source of real-time free radical generation in the tissue.

Previous protocols have described using biotin-HZ to label protein carbonyls in tissue protein extract. However, these methods rely on molecular weight as the only suggestive factor of target protein identity. This current protocol combines immunoreaction with protein purification methods to measure protein carbonylation in a specific target protein more accurately. Nonetheless, the effectiveness of the pulldown or IP assay depends on the abundance of target protein in the object tissue. Proteins with trace expression or those which are minimally carbonylated are sub-optimal for this protocol. In addition, it is ideal to have comparable protein levels between group of samples. Extra caution will be needed if comparing protein carbonylation between samples with variable protein abundance. Lysate control (input) is therefore necessary to ensure the comparison is proportional instead of an outcome of under- or over-expression of the target protein in the experimental group.

## Troubleshooting

### Problem 1

No star cell or axial muscle detection aside strong αSMA stain in the *muscularis externa* in younger tissues from step 9.

### Potential solution

Make sure the tissue is fixed in ice-cold methanol and has never exposed to formaldehyde-based fixatives.

Failure to remove the intestinal contents and pancreatic tissue often leads to tissue degradation during processing. Make sure to remove intestinal contents and pancreatic tissue while keeping the tissues on ice and/or at 4°C throughout the entire whole mount staining protocol.

Given that the αSMA signal of axial muscle and star cells is quite low in younger tissues when compared to the gut wall, it is sometimes necessary to overexpose the muscularis externa signal in imaging for acquiring appropriate αSMA stain details in the *lamina propria*.

Older tissue larger than 0.5 cm^3^ may have poor Ab penetrance. Slice tissues into smaller pieces or take extra steps to permeabilize the tissue by bringing through sucrose/glycerol gradient (Bernier-Latmani and Petrova, 2016).

### Problem 2

Loss of intrinsic fluorescent signals in methanol fixed tissues after intestine isolation step 3.

### Potential solution

In some circumstances, tissues from genetic modified animals carrying intrinsic fluorescent protein expression are used to colocalize αSMA expressions. Methanol fixation will quench the intrinsic fluorescent signal, but this can be overcome by immunofluorescent stain on the intrinsic fluorescent protein of interest. For example, anti-RFP antibody followed by secondary antibody incubation can retrieve transgenic tdTomato signals quenched by methanol.

### Problem 3

Protein extract supernatant too viscous to remove after centrifugation in step 12.

### Potential solution

This is likely caused by excessive genomic DNA contamination in the sample tissues. To overcome this, methods described below are recommended to shear genomic DNA:

Increase period of fine needle pulling (step 1D).

Sonication.

DNase treatment.

### Problem 4

Lack of distinct bands or signal on the blot in step 47.

### Potential solution

Increase protease inhibitor concentration to avoid protein degradation during the procedure if seeing no signals in the lysate control. A baseline level of protein carbonyl should be detected in all tissues regardless of genotypes.

An extended interaction with antibody or streptavidin can be used to increase protein capture. Increased volume, time, and stringency of wash buffer can be used to decrease the presence of bands interpreted as non-specific: for example, signal in streptavidin pulldown from non-biotin-hydrazide treated samples and/or bands outside of the predicted molecular weight of actin (∼42 kDa) in the actin pulldown.

The dilution of primary and/or secondary antibody can be adjusted if no signal is observed or if signal reflects an excess of what is interpreted as non-specific bands.

### Problem 5

Inefficient pulldown or IP.

### Potential solution

Protein carbonyls detected in Streptavidin pulldown supernatant in step 47.

Adjust the ratio of sample to streptavidin beads by reducing sample amount or increasing streptavidin beads amount.

Increase the incubation time for the sample/streptavidin beads mixture.

Smooth muscle actin detected in supernatant from αSMA IP in step 47.

Adjust the ratio of sample to αSMA antibody by reducing sample amount or increasing αSMA antibody amount.

Increase sample/αSMA antibody incubation time.

### Problem 6

Saturated signal on the blot (potentially obscuring differential carbonyl content between samples) in step 47.

### Potential solution

Load less sample on the gel or take a shorter exposure when visualizing protein.

Decrease starting protein amount to keep the reaction within its linear range for proper comparison.

## Resource availability

### Lead contact

Further information and requests for resources and reagents should be directed to and will be fulfilled by the lead contact, Natasza A. Kurpios (natasza.kurpios@cornell.edu).

### Materials availability

This study did not generate new unique reagents.

## Data Availability

This study did not generate/analyze [datasets/code].
